# Tuberculosis and Takayasu arteritis: a case report 

**DOI:** 10.1186/s13256-023-04037-2

**Published:** 2023-07-17

**Authors:** Maryem Ferjani, Mounira El Euch, Mariem Boumediene, Mariem Jrad, Fethi Ben Hamida, Sami Turki, Tahar Gargah

**Affiliations:** 1 Pediatric Department, Charles Nicolle Hospital of Tunis, Tunis, Tunisia; 2 Internal Medicine Department “A”, Research Laboratory of Kidney Diseases (LR00SP01), Boulevard 9 Avril, Bab Souika, 1006 Tunis, Tunisia; 3 Department of Imaging, Charles Nicolle Hospital of Tunis, Tunis, Tunisia; 4 Medicine Faculty of Tunis, University of Tunis El Manar, Tunis, Tunisia

**Keywords:** Takayasu arteritis, Tuberculosis, Child, Case report

## Abstract

**Background:**

Takayasu arteritis is a rare and chronic granulomatous vasculitis that affects the large vessels. Takayasu arteritis targets the aorta and its branches and is still of unknown etiology. It often affects female patients under 50 years of age. A relationship between Takayasu arteritis and tuberculosis has been suggested for a long time.

**Case presentation:**

We report a severe case of Takayasu arteritis in a 10-year-old Tunisian child revealed by renovascular hypertension with concomitant pulmonary tuberculosis.

**Conclusions:**

Our patient is among only a few cases of Takayasu arteritis published worldwide affecting young infants and adolescents, which underlines the strong relationship between Takayasu arteritis and tuberculosis.

## Background

Takayasu arteritis (TAK) is a chronic granulomatous vasculitis of large vessels that affects the aorta and its main branches. This granulomatous inflammation may lead to stenosis, occlusion, dilatation, or aneurysm of the involved arteries [1]. Although TAK is frequently seen in young women, it can also affect young infants and adolescents [2]. TAK in childhood is rare, with only a few cases of childhood-onset TAK reported in literature until now [3].

Features are variable depending on the stage of disease. In fact, the first stage of initial inflammatory process is often unrecognized and characterized by systemic signs. In the second stage, multiple arterial occlusions and stenosis can occur, and can be revealed by signs of cerebral, visceral, or extremity ischemia [4]. In childhood, the lack of specificity of first symptoms in the first stage may extend and make diagnosis difficult and treatment too late [5]. In these conditions, radiological findings can be helpful in making the correct diagnosis.

Etiopathogenesis of TAK remains hypothetical, and is hypothesized to be related to genetics, endocrine abnormalities, and infections such as *Mycobacterium tuberculosis* (TB) [6]. In fact, both latent and active TB infection have been observed in patients with TAK [7].

This report describes the case of a young girl with extensive TAK concomitant to pulmonary TB.

## Case presentation

A 10-year-old Tunisian girl presented to our hospital with prolonged fever during 2 months. Her parents were first-degree relatives and she had no past medical history. She received the Bacillus Calmette-Guerin (BCG) vaccine as a child and had no prior contact with a family member with TB infection. On physical examination, there was generalized butterfly-rash-like erythematous eruption, high fever, and arthralgia. Blood pressure measurement revealed hypertension with different levels between the two upper limbs (145/80 mmHg on the left arm and 160/90 mmHg on the right arm). Both carotid left artery and left para umbilical bruits were assessed and palpation of all peripheral pulses was normal. She had no lymphadenopathy, splenomegaly, or hepatomegaly, and dipstick test was normal. Labstix strip test was negative.

**Fig. 1:**
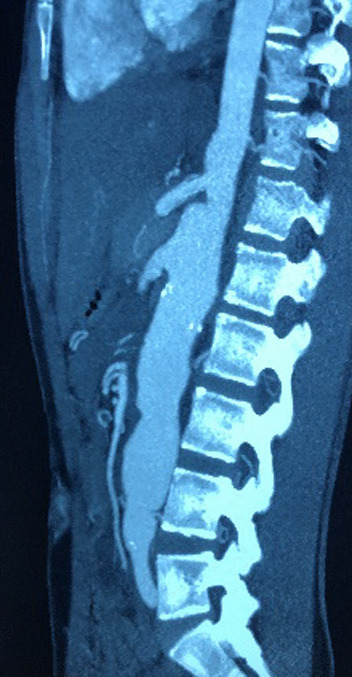
Computed tomography angiography showing diffuse artery thickening with luminal irregularity, inducing multiple stenosis and aneurysms, located in the aorta and its branches, aortic arch, descending thoracic, and abdominal aorta up to its infrarenal segments

**Fig. 2:**
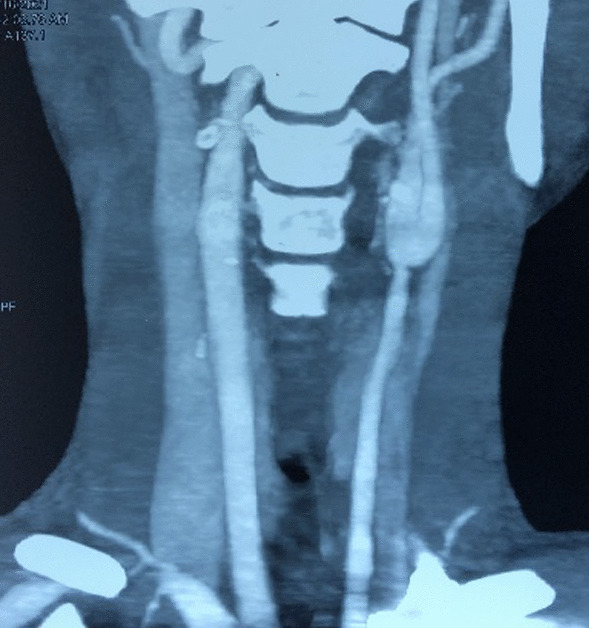
Left common carotid artery abnormalities as assessed on computed tomography angiography

**Fig. 3:**
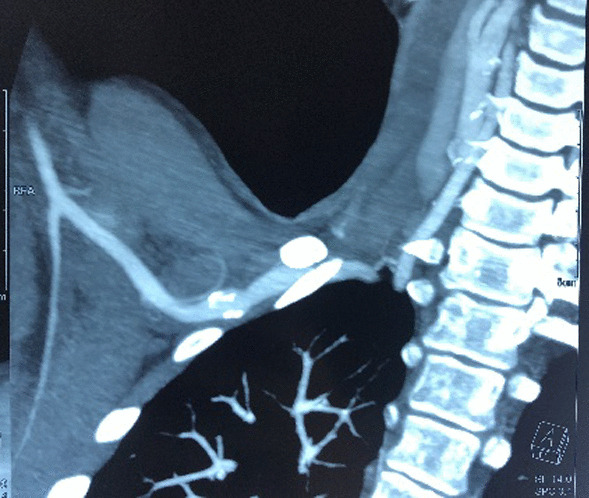
Right subclavian artery abnormalities on computed tomography angiography

**Fig. 4:**
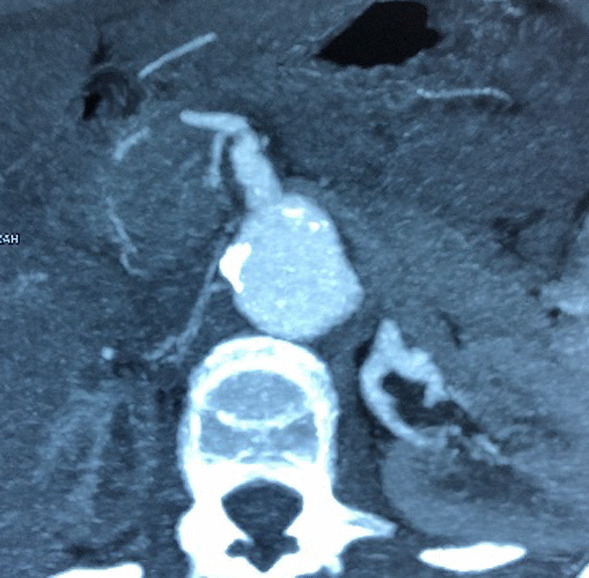
Celiac trunk, superior mesenteric artery and the two renal arteries abnormalities on computed tomography angiography

Laboratory tests showed microcytic anemia (hemoglobin at 9.5 g/dl) with normal leukocytes and platelets counts. The C-reactive protein was at 82 mg/l and erythrocyte sedimentation rate was elevated at 118 mm/hour. Other analyses were within normal reference ranges as following: urea 2.9 mmol/l, creatininemia 37 μmol/l, sodium 136 mmol/l, potassium 4.5 mmol/l, and calcium 2.45 mmol/l. Repeated blood cultures were negative. Protein electrophoresis revealed elevated alpha-2 and gamma globulins without nephrotic syndrome. Serum ferritin, fibrinogen, and liver function tests were normal. Immunological tests including rheumatoid factor, anti-nuclear antibodies complement components C3 and C4, and antineutrophil cytoplasmic were negative. Viral serologies, including coronavirus disease 2019 (COVID-19), human immunodeficiency virus (HIV), cytomegalovirus, Epstein–Barr virus, herpes, parvovirus, and hepatitis C and B, were negative. Bone marrow aspiration showed no significant abnormalities. Both Quantiferon-TB-Gold in Tube and tuberculin skin tests were positive. Chest and knee X-rays were normal. BK detection in bronchial sputum was positive to mycobacterium TB. Chest computed tomography (CT) scan revealed alveolar syndrome in the upper left lobe. Electrocardiogram showed signs of left ventricular hypertrophy, and transthoracic echocardiography was normal. Ophthalmological examination was unremarkable. Renal Doppler ultrasound showed a small right kidney with increased circulation velocities in the left renal artery.The cervical Doppler ultrasonography showed a low flow of the right subclavian artery and a circumferential and stenotic thickening of the left carotid artery. CT angiography showed diffuse artery thickening with luminal irregularity, inducing multiple stenosis and aneurysms, located in the aorta and its branches, aortic arch, descending thoracic, and abdominal aorta up to its infrarenal segments (Fig. 1), left common carotid artery (Fig. 2), right subclavian artery (Fig. 3), celiac trunk, superior mesenteric artery, and the two renal arteries (Figs. 1 and 4).

TAK diagnosis concomitant to TB infection was diagnosed and we prescribed a four-drug anti-TB regimen including rifampin, isoniazid, pyrazinamide, and etambutol during 2 months. We then maintained only rifampicin and isoniazid in association to methyl prednisolone pulses of 1 g/day during 3 days relayed by prednisone at 1 mg/kg/day. In addition to corticosteroid treatment, the patient received methotrexate at 10 mg/m^2^/week with folic acid, calcium, and vitamin D. Although inflammatory biological syndrome and anemia vanished, hypertension was not easy to control and required a combination of three drugs: calcium channel blockers, beta blockers, and alpha blockers. Follow-up was favorable clinically, biologically, and radiologically.

## Conclusions

Although the exact incidence of TAK in childhood has not been exactly determined until now, it seems to be a rare disease due partly to the lack of awareness of primary care physicians and the huge variety of its clinical presentations [8]. Our patient is the second case of TAK diagnosed in our department [9] and among only a few cases of TAK published worldwide affecting young infants and adolescents [10].

TAK diagnosis was suspected regarding clinical findings such as fever and hypertension, which are the most reported in literature[11] and confirmed by radiological features since CT angiography is among the gold standard of radiological exploration [12]. Our patient fulfilled criteria of TAK according to the most scientific societies [European League Against Rheumatism (EULAR)/Paediatric Rheumatology International Trials Organisation (PRINTO)/Paediatric Rheumatology European Society (PRES) for pediatric patients, and American College of Rheumatology] [13, 14]. Despite its rarity, TAK is more common in countries with high incidence of TB, such as Tunisia [15]. The relationship between TAK and TB is still unknown and some studies underlined the similarity of giant Langerhans-like cells and granulomas both in TAK and TB [16]. Other studies showed that the humoral and cellular immunity in a group of patients with TAK were similar to host responses to Mycobacterium TB infection [17]. Indeed, some authors suggested that Mycobacterium TB could take advantage of the high concentration of oxygen in the aorta, evade the immune system, and proliferate in its wall [18], which would explain how arteritis might be the result of a TB infection directly in the vessel wall. This association is more frequent after anti-tumor necrosis factor alpha (TNFα) inhibitor therapy in patients with TAK, with increased risk up to 25 times induced by reactivation and dissemination of Mycobacterium TB from latent foci of infection [19]. Lungs are the most frequent anatomic site of TB infection in TAK, as in our patient [16]. Fortunately, she did not develop severe complications such as dissecting aneurysm, ocular, or skin TB complications reported in some cases [20–22]. We insist on the importance of focusing on and treating TB infections before starting corticosteroids and immunosuppressive treatment mainly in countries with high prevalence of TB [23]. Although there is no evidence that anti-TB therapy prevents TAK progression or its complications, the combination of corticosteroids and anti-TB drugs was efficient to control the disease activity observed in our patient [24].Glucocorticoids should be combined with immunosuppressive drugs including methotrexate, cyclophosphamide, or mycophenolate mofetil to control disease activity [15]. Biological therapies such as TNFα, anti‐interleukin‐6 agents, rituximab, and tocilizumab seem to be promising in case of lack of response to first-line treatment [25]. Finally, difficulties in controlling hypertension in our patient could be explained by renal artery stenosis, since endovascular treatment was not possible because of the long-segment stenosis. In case of persistent hypertension, unilateral nephrectomy could be discussed.

TAK in children remains a challenging diagnosis because of nonspecific features. We insist that TAK must be suspected when regarding systemic signs with hypertension and/or blood pressure differences between extremities. A strong relationship between TAK and TB is established and anti-TB drugs must be initiated before corticosteroid treatment. A causal relationship between TB and TAK and evidence of prevention of TAK progression and complications under anti-TB therapy need further investigation. In TAK, a close monitoring of the clinical disease activity and damage associated with inflammatory markers and imaging are necessary to better identify the onset of disease and adapt therapy to prevent morbidity and mortality. The presence of chronic medical conditions can worsen the prognosis of these conditions [26, 27].

## Data Availability

All information are available in medical file’s patient in pediatrics department of Charles Nicolle hospital, Tunis, Tunisia.
